# A response to Al et al. Trials 2023;24:233

**DOI:** 10.1186/s13063-023-07574-9

**Published:** 2023-08-13

**Authors:** Jon F. Merz

**Affiliations:** Department of Medical Ethics & Health Policy, Perelman School of Medicine at the University of Pennsylvania, Blockley Hall 1427, 423 Guardian Drive, Philadelphia, PA 19104-4884 USA

**Keywords:** Informed consent, Refusal, Psychological manipulation, Voluntariness

## Abstract

In their recent paper, Al and colleagues (Trials 2023;24:233) argue that manipulation of the methods of recruitment using well-known techniques in order to increase enrollment can be ethically acceptable. This brief response challenges that notion as an affront to voluntariness and a devolution of the ethics of human subjects research to the “ethics” of the marketplace.

To the Editor:

In their recent paper, Al and colleagues [1] argue that, at least with nonvulnerable subjects, manipulation of the methods of recruitment using well-known techniques in order to increase enrollment can be ethically acceptable. They do add that researchers attempting to use such methods should study their practices (perhaps to ensure they do not rise to the level of coercing or unduly influencing potential subjects), and disclose their methods and purpose to relevant ethics committees. The examples they provide demonstrate the use of manipulation of information (setting social norms, framing of benefits and losses), and “leveraging” the trust patients place in their physicians to bolster recruitment.

The paper appears to be premised on a strong assumption that research participation is the correct normative choice for potential subjects and that researchers (and ethics review boards) are therefore justified in purposefully using psychological manipulation to influence potential subjects’ decisions, biasing people toward participation. If we start from an opposite assumption, say one in which subjects are to be protected from research risks, then ethics boards “should” be promoting methods to dissuade potential subjects from taking part. In my opinion, neither assumption is wholly correct, so methods having the express purpose and intent of biasing people one way or the other seem illegitimate and dangerous.

A review of reviews conducted several years ago suggested that, across a broad range of human subjects research, something on the order of 30% of potential subjects refuse to participate, ranging from zero to nearly 100% [2]. This rate of refusal is not shocking to established reseachers. Perhaps the descriptive should set the norm; I for one worry about recruitment methods when researchers report no or few refusals. Having served on 3 different Institutional Review Boards, I worried too when I heard reviewers conclude that they personally wouldn’t participate, but if “patients” are willing to take part, they won’t stand in their way. Informed consent is imperfect, but putting researchers’ and ethics reviewers’ hands on the scale to tip the balance is paternalism run amok and presents a serious risk to voluntariness [3, 4].

Al et al. justify the use of manipulative methods in part on the basis that “behavioral influence strategies could be considered prima facie autonomy-respecting if it is comparable to other uncontroversial behavioral influence strategies to which an individual is routinely exposed in their daily life,” (pg. 4) drawing an analogy to minimal risk, “daily-life” standards for waiver or modification of informed consent. We citizens are bombarded on a daily basis with multimedia marketing and targeted advertising, all of which draw on the well-established tools of propaganda [5–7]. I for one hope that research ethics will not devolve to the “ethics” of the marketplace. Moreover, while it might be argued that these methods might be used simply to enhance the likelihood of potential subjects paying attention and hearing or reading a research pitch (or consent form), people are completely free to give “uninformed refusals.” [8].

I suggest that a better approach is to try to help potential subjects make the best decisions they can under the circumstances, drawing on their own values, hopes, and tolerances for risk and uncertainty. This may increase or decrease recruitment, but in the end, research should serve the interests of subjects as much as, if not more than, other stakeholders, because we ask the most of subjects — they have the most to lose and not necessarily the most to gain.

## Data Availability

Not applicable.

## References

[1] Al P, Hey S, Weijer C, Gillies K, McCleary N, Yee ML, Inglis J (2023). Changing patient preferences toward better trial recruitment: an ethical analysis. Trials.

[2] Baker FX, Merz JF (2018). What gives them the right? legal privilege and waivers of consent for research. Clin Trials.

[3] Nelson RM, Merz JF (2002). Voluntariness of consent for research: an empirical and conceptual review. Med Care.

[4] Appelbaum PS, Lidz CW, Klitzman R (2009). Voluntariness of consent to research: a conceptual model. Hastings Cent Rep.

[5] Stanley J (2015). How propaganda works.

[6] Rutherford P (2000). Introduction: advertising as propaganda. In: Endless propaganda: the advertising of public goods.

[7] Noddings N (2006). Advertising and propaganda. In: Critical lessons: what our schools should teach..

[8] Vorholt V, Dickert NW (2019). Uninformed refusals: objections to enrolment in clinical trials conducted under an Exception from Informed Consent for emergency research. J Med Ethics.

